# ETHICAL DILEMMAS, VULNERABLE ELDERS, AND ELDER ABUSE

**DOI:** 10.1093/geroni/igz038.891

**Published:** 2019-11-08

**Authors:** Candace J Heisler

**Affiliations:** Heisler and Associates, San Bruno, California, United States

## Abstract

Elder abuse is a growing concern worldwide. It is described across multiple professional disciplines: as a social justice issue by social workers; as a medical syndrome and public health issue by health care providers; and as a violation of human rights and criminal laws by courts, legislators, and the justice system professionals. Elder abuse assumes different forms, including physical, emotional, and sexual abuse, financial exploitation, neglect, and abandonment. Forms often co-occur in a variety of settings. This presentation explores key ethical conundrums emerging when different professions address elder abuse. Specifically examined is how the ethical principles of autonomy and non-maleficence conflict with mandatory reporting laws, for example, if their purpose is to incarcerate older offenders who are ill and vulnerable serving lengthy mandated prison terms. The presentation also explores the rights of perpetrators, including how rights of crime victims are weighed against those of perpetrators in a just society.

